# Ganoderic Acid A Promotes Amyloid-β Clearance (In Vitro) and Ameliorates Cognitive Deficiency in Alzheimer’s Disease (Mouse Model) through Autophagy Induced by Activating Axl

**DOI:** 10.3390/ijms22115559

**Published:** 2021-05-24

**Authors:** Li-Feng-Rong Qi, Shuai Liu, Yu-Ci Liu, Ping Li, Xiaojun Xu

**Affiliations:** 1 State Key Laboratory of Natural Medicines, China Pharmaceutical University, Nanjing 210009, China; 1831070172@stu.cpu.edu.cn (L.-F.-R.Q.); ls1149074157@yahoo.com (S.L.); 3320071813@stu.cpu.edu.cn (Y.-C.L.); liping2004@126.com (P.L.); 2 Jiangsu Key Laboratory of Drug Discovery for Metabolic Diseases, China Pharmaceutical University, Nanjing 210009, China

**Keywords:** Alzheimer’s disease, amyloid β, ganoderic acid A, autophagy, Axl

## Abstract

Alzheimer’s disease (AD) is thought to be caused by amyloid-β (Aβ) accumulation in the central nervous system due to deficient clearance. The aim of the present study was to investigate the effect of ganoderic acid A (GAA) on Aβ clearance in microglia and its anti-AD activity. Aβ degradation in BV2 microglial cells was determined using an intracellular Aβ clearance assay. GAA stimulated autophagosome formation via the Axl receptor tyrosine kinase (Axl)/RAC/CDC42-activated kinase 1 (Pak1) pathway was determined by Western blot analyses, and fluorescence-labeled Aβ42 was localized in lysosomes in confocal laser microscopy images. The in vivo anti-AD activity of GAA was evaluated by object recognition and Morris water maze (MWM) tests in an AD mouse model following intracerebroventricular injection of aggregated Aβ42. The autophagy level in the hippocampus was assayed by immunohistochemical assessment against microtubule-associated proteins 1A/1B light-chain 3B (LC3B). Intracellular Aβ42 levels were significantly reduced by GAA treatment in microglial cells. Additionally, GAA activated autophagy according to increased LC3B-II levels, with this increased autophagy stimulated by upregulating Axl and Pak1 phosphorylation. The effect of eliminating Aβ by GAA through autophagy was reversed by R428, an Axl inhibitor, or IPA-3, a Pak1 inhibitor. Consistent with the cell-based assay, GAA ameliorated cognitive deficiency and reduced Aβ42 levels in an AD mouse model. Furthermore, LC3B expression in the hippocampus was up-regulated by GAA treatment, with these GAA-specific effects abolished by R428. GAA promoted Aβ clearance by enhancing autophagy via the Axl/Pak1 signaling pathway in microglial cells and ameliorated cognitive deficiency in an AD mouse model.

## 1. Introduction

Alzheimer’s disease (AD) is a progressive and nonreversible disorder characterized by impairment of memory and cognition. According to recent reports, AD represents a major public health concern and has been identified as a research focus [1]. Aggregation of amyloid-β (Aβ)42, the primary component of senile plaques, is the critical factor of AD pathology. The amyloid hypothesis assumes that the Aβ peptide is the causative agent in AD and is strongly supported by data from rare autosomal dominant forms of AD [2]. Moreover, numerous studies demonstrate that AD might be caused by mutations in three genes (*APP*, *PSEN1*, and *PSEN2*), involved in Aβ production [3]. Despite significant efforts to reduce Aβ generation, the field has seen little therapeutic success [4,5]. The development of late-onset AD appears to arise from the failure of Aβ-clearance mechanisms rather than overproduction of the peptide [6]. The clearance of intracellular Aβ occurs through proteolysis by a family of amyloid-degrading enzymes (ADEs) or through other degradation processes, such as autophagy [7,8]. ADEs include the neprilysin family, insulin-degrading enzyme, and angiotensin converting enzyme [9,10,11]. Early activation of microglia also facilitates Aβ clearance by phagocytosis [12]. Therefore, activation of microglia can be considered a good therapeutic strategy to degrade and remove Aβ to relieve AD symptoms.

Axl is expressed in the adult central nervous system (CNS), particularly in the cerebellum and hippocampus [13]. Emerging evidence establishes Axl as both a controller of microglial physiology and a potential therapeutic target for central CNS [14,15,16]. Compared with wild-type mice, there were fewer activated microglia surrounding lesions in *Axl*^−/−^ mice, suggesting that the loss of Axl affected migration of microglia to the injury site to restrict the damage and clear cell debris [17]. Our previous study found that Axl activation stabilized the heat shock protein 90β/peroxisome proliferator-activated receptor-γ complex to promote expression of Aβ42-clearance-related genes, including *ApoE*, *Abcg1*, and *Abca1* [18].

Autophagy is a highly conserved homeostatic degradation process in eukaryotic cells and plays an important role in cell survival and maintenance through the degradation of cytoplasmic organelles, proteins, and macromolecules via lysosomal compartments [19]. Enhanced autophagic activity reportedly promotes Aβ clearance in some models [7,20]. Although several key regulators of macroautophagy, such as AMP-activated protein kinase and mammalian target of rapamycin complex 1 [19], have been identified, it is likely that many regulatory factors have not yet been defined. A previous report suggested that induction of Gas6-Axl signaling-mediated autophagy in murine macrophages ameliorates hepatic inflammatory responses [21]. Moreover, findings indicate that Axl mediates the activation of the small GTPase Rac1 [15,22], which improves post-stroke recovery and angiogenesis mediated by Pak1 [23]. Pak1 also plays an instrumental role in initiating hypoxia-induced autophagy and maintaining glioblastoma growth [24]; however, whether Axl/Pak1 signaling plays a role in regulating autophagy remains unknown.

*Ganoderma lucidum* is a multi-purpose plant medicine that is homologous to functional food. The pharmacological activities of *G. lucidum*, particularly its intrinsic immunomodulating and antitumor properties, have been well-documented [25]. Ganoderma is reportedly as a treatment for neurodegenerative diseases [26,27]. Ganoderic acid A (GAA), a highly oxygenated tetracyclic triterpenoid, is the major active component of *Ganoderma lucidum* [28]. Most *G. lucidum* triterpenoids (GLTs) exhibit extensive bioactivities, including anticancer, antihypertensive, immunomodulatory, antioxidant, and neuroprotective effects [26,29,30]. In the present study, we investigated the role of GAA in Aβ42 clearance and autophagy using microglial cells. Furthermore, in vivo anti-AD activity of GAA was evaluated in an AD mouse model intracerebroventricularly (i.c.v.) injected with aggregated Aβ42. These findings might be beneficial to providing new strategies for identifying potential drugs for treating AD.

## 2. Results

### 2.1. GAA Facilitates Aβ42 Degradation In Vitro BV2 Microglial Cells

To investigate whether *Ganoderma* extract can promote Aβ degradation in BV2 cells, we enriched and purified GLTs from the ethyl acetate layer and *G. lucidum* polysaccharides (GLPs) from the water layer of *G. lucidum* extract (Figure S1A). Among the GLTs, we detected GAA (Figure S1B), GAD (Figure S1C), and other components (Figure S1D) by MS.

**Figure 1:**
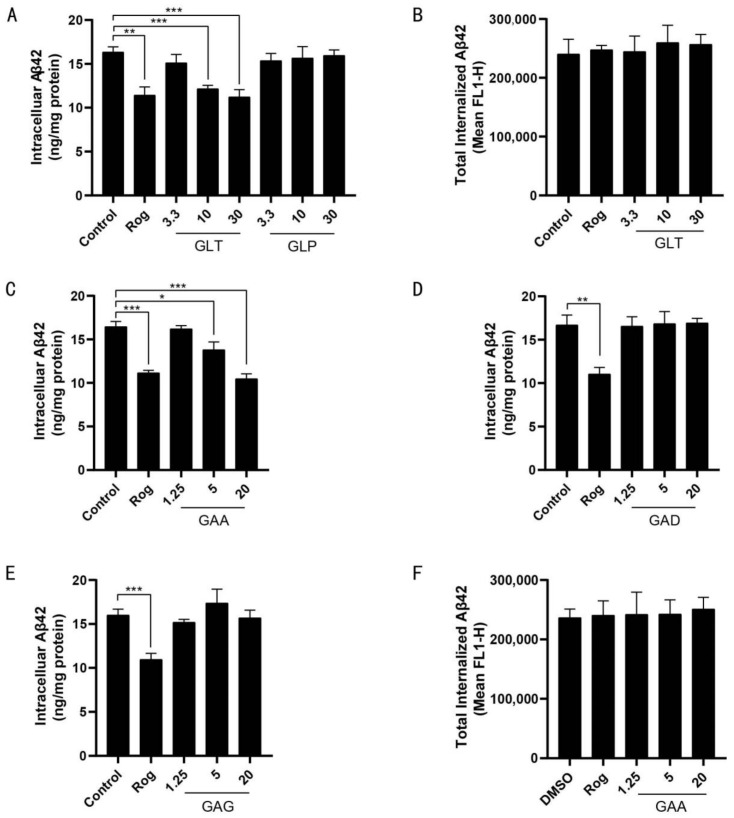
GAA facilitates Aβ42 degradation in microglial cells. (**A**) BV2 cells were treated with Rog (10 µM), GLT, and GLP at the indicated concentrations in the presence of Aβ42 (2 µM) for 24 h, and intracellular Aβ42 levels were measured using ELISA. Rog vs. Control: *p* = 0.0016; GLT-10 vs. Control: *p* =0.0005; GLT-30 vs. Control: *p* = 0.001; *n* = 3. (**B**) Aβ42 uptake by BV2 was assessed by applying FITC-labeled Aβ42 (1 µM) to BV2 cells in the presence of Rog (10 µM) and GLT at the indicated concentrations for 24 h. Accumulation of the fluorophore was analyzed by flow cytometry. *n* = 3. (**C**–**E**) BV2 cells were treated with Rog (10 µM), GAA, GAD, and GAG, respectively, at the indicated concentrations in the presence of Aβ42 (2 µM) for 24 h, and intracellular Aβ42 levels were measured using ELISA. (**C**) Rog vs. Control: *p* = 0.0002; GAA-5 vs. Control: *p* = 0.0132; GAA-20 vs. Control: 0.0002; *n* = 3. (**D**) Rog vs. Control: *p* = 0.0021; *n* = 3. (**E**) Rog vs. Control: *p* = 0.0009; *n* = 3. (**F**) Uptake of Aβ42 by BV2 was assessed by applying FITC-labeled Aβ42 (1 µM) to BV2 cells in the presence of Rog (10 µM) and GAA at the indicated concentrations for 24 h, and accumulation of the fluorophore was analyzed by fluorescence signal 1-height (FL1-H) of flow cytometry. Results were normalized to total cellular protein level, and DMSO at 0.1% was used as the control. *n* = 3. *n* is the number of replicates (biological and technical) used for each of the described results. * *p* < 0.05, ** *p* < 0.01, *** *p* < 0.001 vs. indicated control.

GLT treatment significantly reduced intracellular Aβ42 levels in BV2 cells (Figure 1A), whereas GLP did not show such an effect, suggesting that the major active components involved in reducing intracellular Aβ42 were GLTs. FITC-labeled Aβ42 is taken up and degraded by microglia, and after Aβ42 degradation, the fluorophore remains in the cells; therefore, total cellular fluorescence correlates to total Aβ42 uptake and can be monitored by flow cytometry [31]. To exclude the possibility that decreased Aβ42 is due to reduced uptake, flow cytometry and FITC-labeled Aβ42 were used to monitor the cumulative internalization of Aβ42. Total internalized Aβ42 levels in BV2 cells were unaffected by GLT treatment (Figure 1B). We then assayed the effect of each GLT component by measuring intracellular Aβ42 levels in BV2 cells. Only GAA and neither GAD nor GAG treatment significantly reduced intracellular Aβ42 levels in BV2 cells (Figure 1C–E), with no observed effect by GAA on total internalized Aβ42 levels in BV2 (Figure 1F). These data suggested that GAA facilitates Aβ42 degradation in microglial cells.

### 2.2. GAA Promotes Aβ42 Elimination through the Autophagy Pathway In Vitro BV2 Microglial Cells

**Figure 2:**
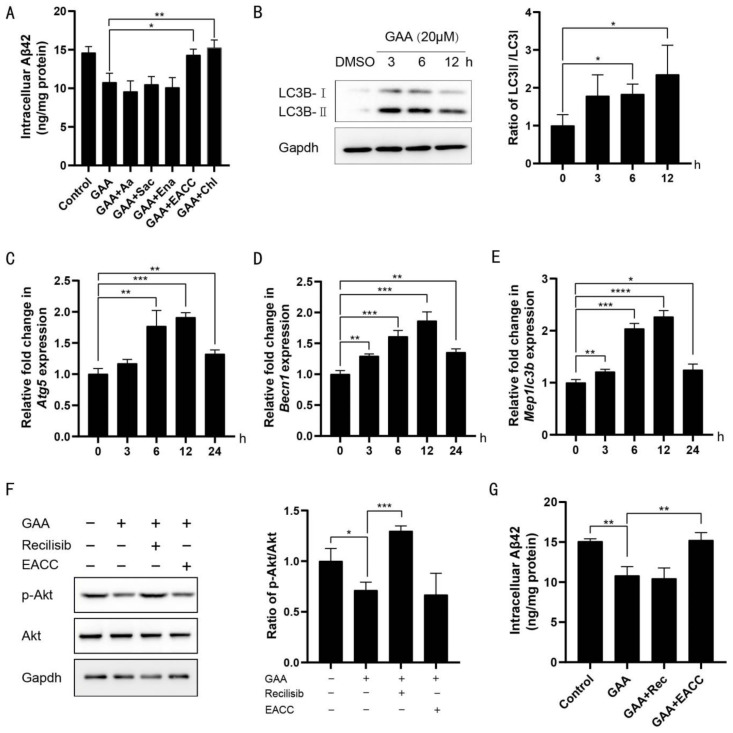
GAA promotes Aβ42 elimination through the autophagy pathway in microglial cells. (**A**) BV2 cells were treated with GAA (20 µM), the indicated antagonists of ADEs (arachidonic acid at 80 µM, sacubitrilat at 50 µM, enalapril maleate at 50 µM), autophagosomes (EACC at 2 µM) and lysosomes (chloroquine at 20 µM), in the presence of Aβ42 (2 µM) for 24 h, and intracellular Aβ42 levels were measured by ELISA. GAA + EACC vs. GAA: *p* = 0.0122; GAA + Chl vs. GAA: *p* = 0.0075; *n* = 3. (**B**) BV2 cells were treated with GAA (20 µM) for the indicated periods of time, and whole cell proteins were collected for western blot. 6 h vs. 0 h: *p* = 0.0219; 12 h vs. 0 h: *p* = 0.0467; *n* = 3. (**C**–**E**) BV2 cells were treated with GAA (20 µM) for the indicated times, and expression of *Atg5, Becn1* and *Map1lc3b* was analyzed by qRT-PCR. (**C**) 6 h vs. 0 h: *p* = 0.0075; 12 h vs. 0 h: *p* = 0.0002; 24 h vs. 0 h: *p* = 0.0069; *n* = 3. (**D**) 3 h vs. 0 h: *p* = 0.0015; 6 h vs. 0 h: *p* = 0.0007; 12 h vs. 0 h: *p* = 0.0006; 24 h vs. 0 h: *p* = 0.0017; *n* = 3. (**E**) 3 h vs. 0: *p* = 0.0086; 6 h vs. 0 h: *p* = 0.0001; 12 h vs. 0 h: *p* < 0.0001; 24 h vs. 0 h: *p* = 0.0286; *n* = 3. (**F**) BV2 cells were treated with GAA (20 µM), the indicated agonist of AKT/PI3K (recilisib at 20 µM) and EACC (2 µM) for 6 h, and whole cell proteins were collected for western blot. GAA vs. Control: *p* = 0.0288; GAA + Recilisib vs. GAA: *p* = 0.0004; *n* = 3. (**G**) BV2 cells were treated with GAA (20 µM), recilisib (20 µM) and EACC (2 µM), in the presence of Aβ42 (2 µM) for 24 h, and intracellular Aβ42 levels were measured by ELISA. DMSO at 0.1% was used as the control. GAA vs. Control: *p* = 0.0031; GAA + EACC vs. GAA: *p* = 0.0064; *n* = 3. *n* is the number of replicates (biological and technical) used for each of the described results. * *p* < 0.05, ** *p* < 0.01, *** *p* < 0.001, **** *p* < 0.0001 vs. indicated control.

To determine which enzyme plays a pivotal role in degrading Aβ, we applied inhibitors of these ADEs. Arachidonic acid, sacubitrilat, and enalapril, which inhibit insulin-degrading enzyme, neprilysin, and angiotensin converting enzyme [32,33,34], respectively, did not affect GAA-induced Aβ clearance in BV2 cells. By contrast, both the autophagy antagonist EACC and the lysosome antagonist chloroquine impeded Aβ degradation [35,36] (Figure 2A), suggesting that the autophagosome/lysosome pathway is essential for GAA-induced Aβ clearance.

To confirm these results, we measured changes in the ratio of LC3B-I to LC3B-II, as conversion of LC3B-I to LC3B-II correlates with autophagosome-formations [37]. In agreement with this correlation, GAA-treated BV2 cells showed increased conversion of LC3B-I to LC3BII (Figure 2B). Additionally, in GAA-treated BV2 cells, we observed the induction of autophagy-related genes, including *autophagy-related 5* (*Atg5*), *beclin 1* (*Becn1*), and *microtubule-associated proteins 1A/1B LC3B* (*Map1lc3b*) (Figure 2C–E). Moreover, GAA-treated BV2 cells showed no effect on the expression of LAMP1 as a lysosomal marker (Figure S2A) but decreased the expression of p62 (Figure S2B). Furthermore, to evaluate whether GAA might encourage autophagy by inactivating the phosphoinositide 3-kinase (PI3K)/AKT signaling pathway [38], we found that activating the AKT pathway with recilisib showed no effect on the Aβ-scavenging ability of GAA in BV2 cells (Figure 2F,G). These results demonstrated that GAA stimulates the autophagy pathway to eliminate Aβ42 in BV2 cells independent of inactivation of the PI3K/AKT signaling pathway.

### 2.3. GAA Activates the Autophagy Pathway through Axl In Vitro BV2 Microglial Cells

**Figure 3:**
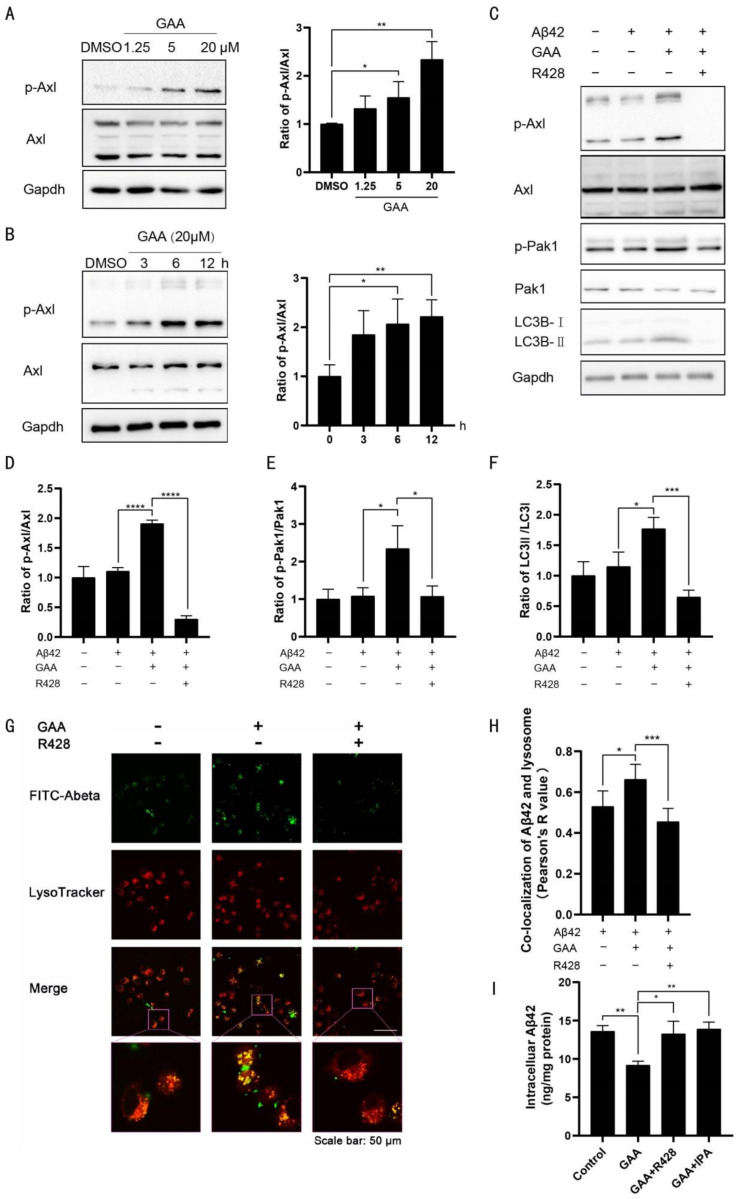
GAA activates the autophagy pathway through Axl. BV2 cells were (**A**) treated with GAA at the indicated concentrations for 6 h or (**B**) treated with GAA (20 µM) for the indicated periods of time, and whole cell proteins were collected for Western blot. (**A**) GAA-5 vs. DMSO: *p* = 0.0452; GAA-20 vs. DMSO: *p* = 0.0034; *n* = 3. (**B**) 6 h vs. 0 h: *p* = 0.0299; 12 h vs. 0 h: *p* = 0.0073; *n* = 3. (**C**–**F**) BV2 cells were pretreated with 0.1% DMSO or the indicated Axl antagonist (R428 at 5 µM) for 30 min, followed by administration of GAA (20 µM) and Aβ42 (2 µM) for 6 h. Whole cell proteins then were collected for Western blot. (**D**) Aβ42 + GAA vs. Aβ42: *p* <0.0001; Aβ42 + GAA + R428 vs. Aβ42 + GAA: *p* < 0.0001; *n* = 3. (**E**) Aβ42 + GAA vs. Aβ42: *p* = 0.028; Aβ42 + GAA + R428 vs. Aβ42 + GAA: *p* = 0.0304; *n* = 3. (**F**) Aβ42 + GAA vs. Aβ42: *p* = 0.0247; Aβ42 + GAA + R428 vs. Aβ42 + GAA: *p* = 0.0009; *n* = 3. (**G**–**H**) BV2 cells were pretreated with R428 (5 µM) for 30 min, followed by administration of FITC-Aβ42 (1 μM) and GAA (20 µM) for 6 h, and cells were immunostained with LysoTracker Red. Representative images show the co-localization of Aβ42 and lysosomes (yellow). Scale bar: 50 µm. Aβ42 + GAA vs. Aβ42: *p* = 0.0469; Aβ42 + GAA + R428 vs. Aβ42 + GAA *p* = 0.0057; *n* = 4. (**I**) BV2 cells were pretreated with the indicated antagonists of Axl (R428 at 5 µM) and Pak1 (IPA-3 at 20 µM), followed by administration of GAA (20 µM) and Aβ42 (2 µM) for 24 h. The intracellular Aβ42 levels were measured by ELISA. DMSO at 0.1% was used as the control. GAA vs. Control: *p* = 0.0012; GAA + R428 vs. GAA: *p* = 0.0151; GAA + IPA vs. GAA: *p* = 0.0015; *n* = 3. *n* is the number of replicates (biological and technical) used for each of the described results. * *p* < 0.05, ** *p* < 0.01, *** *p* < 0.001, **** *p* < 0.0001 vs. indicated control.

We then evaluated whether GAA affects Axl activity. GAA increased the phosphorylation of Axl in concentration- (Figure 3A) and time-dependent manners (Figure 3B) in BV2 cells. Axl promotes activation of Rac family small GTPase 1(Rac1), and Pak1 serves as a Rac1 target [23]; therefore, we hypothesized that GAA activates autophagy in BV2 cells through the Axl/Pak1 signaling pathway. We found that GAA increased Pak1 phosphorylation in BV2 cells (Figure 3C), and that GAA-specific effects on the phosphorylation of Axl and Pak1, as well as conversion of LC3B-I to LC3B-II, were blocked by R428, an Axl-specific inhibitor [39] (Figure 3C–F).

To investigate the link between autophagy and Aβ42 clearance, FITC-labeled Aβ42 was used in BV2 cells stained with LysoTracker Red fluorescent dye. The merged green fluorescence signal of FITC-Aβ42 with red lysosome-specific fluorescence, as shown by the presence of yellow puncta, indicated that Aβ42 was escorted into lysosomes for degradation. We then measured the relative intensity of yellow fluorescence to evaluate Aβ42 degradation within lysosomes, revealing higher intensity of yellow fluorescence in GAA-treated cells (Figure 3G–H), which was abolished by R428. Furthermore, pretreatment with R428 and IPA-3, a Pak1-specific inhibitor [40], completely reversed GAA-induced upregulation of Aβ42 clearance (Figure 3I), strongly indicating that Axl and Pak1 are involved in GAA activity.

### 2.4. GAA Ameliorates Behavioral Deficits in an Aβ-Injected AD Mouse Model

**Figure 4:**
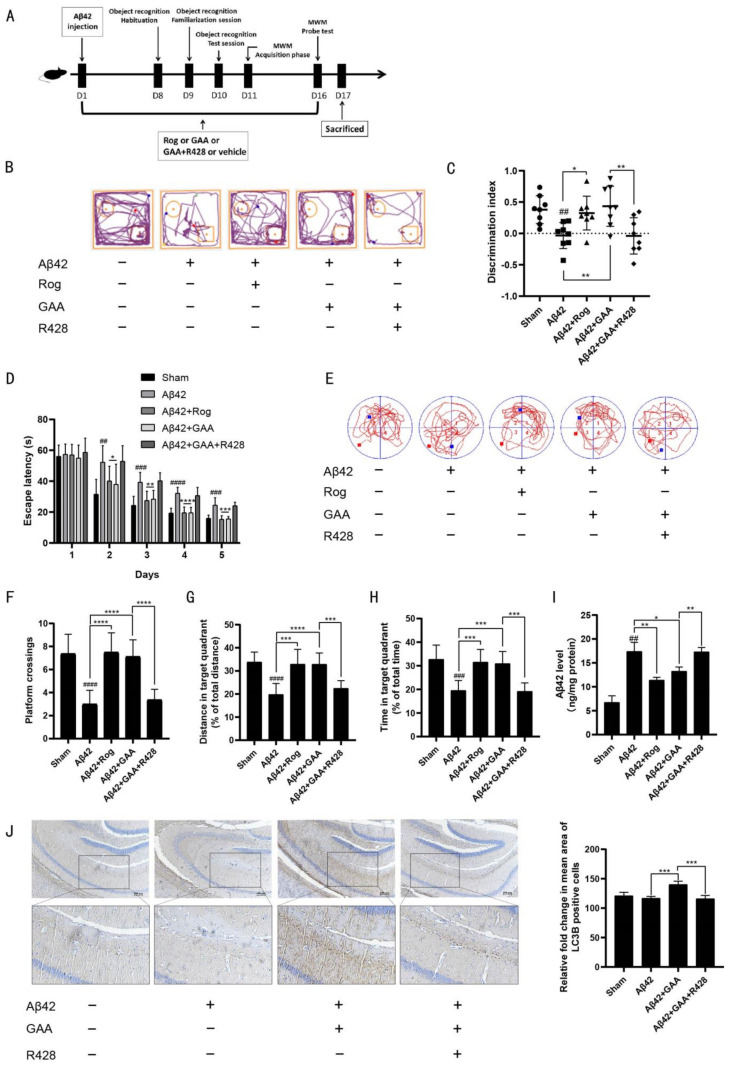
GAA ameliorates Aβ42-induced behavioral deficits in Aβ-injected mice through Axl. (**A**) Experimental design for the animal study. Cerebroventricular injection of aggregated Aβ42 (82 pmol/µL, 5 µL/mouse) was applied to 8-week-old mice. (**B**) Representative motion track and (**C**) the discrimination index in the object recognition test. Aβ42 vs. Sham: *p* = 0.0019; Aβ42 + Rog vs. Aβ42: *p* = 0.0101; Aβ42 + GAA vs. Aβ42: *p* = 0.0038; Aβ42 + GAA + R428 vs. Aβ42 + GAA: *p* = 0.0078; *n* = 8 (**D**) Escape latency during spatial-acquisition training. (Day2) Aβ42 vs. Sham: *p* = 0.0011; Aβ42 + Rog vs. Aβ42: *p* = 0.0303; Aβ42 + GAA vs. Aβ42: *p* = 0.0304; *n* = 8. (Day3) Aβ42 vs. Sham: *p* = 0.0002; Aβ42 + Rog vs. Aβ42: *p* = 0.0019; Aβ42 + GAA vs. Aβ42: *p* = 0.0028; *n* = 8. (Day4) Aβ42 vs. Sham: *p* < 0.0001; Aβ42 + Rog vs. Aβ42: *p* < 0.0001; Aβ42 + GAA vs. Aβ42: *p* < 0.0001; *n* = 8. (Day5) Aβ42 vs. Sham: *p* = 0.0004; Aβ42 + Rog vs. Aβ42: *p* = 0.0002; Aβ42 + GAA vs. Aβ42: *p* = 0.0002; *n* = 8. (**E**) Representative motion track, (**F**) the platform-crossing number, (Aβ42 vs. Sham: *p* < 0.0001; Aβ42 + Rog vs. Aβ42: *p* < 0.0001; Aβ42 + GAA vs. Aβ42: *p* < 0.0001; Aβ42 + GAA + R428 vs. Aβ42 + GAA: *p* < 0.0001; *n* = 8.) (**G**) distance in the target quadrant (Aβ42 vs. Sham: *p* < 0.0001; Aβ42 + Rog vs. Aβ42: *p* = 0.0004; Aβ42 + GAA vs. Aβ42 *p* < 0.0001; Aβ42 + GAA + R428 vs. Aβ42 + GAA: *p* = 0.0002; *n* = 8.) and (**H**) time spent in the target quadrant (Aβ42 vs. Sham: *p* = 0.0002; Aβ42 + Rog vs. Aβ42: *p* = 0.0002; Aβ42 + GAA vs. Aβ42: *p* =0.0003; Aβ42 + GAA + R428 vs. Aβ42 + GAA: *p* = 0.0001; *n* = 8.) in the spatial-probe test. (**I**) Aβ42 level in the hippocampus detected by ELISA. Aβ42 vs. Sham: *p* = 0.0014; Aβ42 + Rog vs. Aβ42: *p* = 0.0064; Aβ42 + GAA vs. Aβ42: *p* = 0.0266; Aβ42 + GAA + R428 vs. Aβ42 + GAA: *p* = 0.0053; *n* = 8. (**J**) LC3B level in the hippocampus detected by IHC assessment. Images were obtained under a microscope (scale bar: 100 μm). Aβ42 + GAA vs. Aβ42: *p* = 0.0004; Aβ42 + GAA + R428 vs. Aβ42 + GAA: *p* = 0.0009; *n* = 4. *n* is the number of replicates (biological and technical) used for each of the described results. ## *p* < 0.01, ### *p* < 0.001, #### *p* < 0.0001 vs. Sham group. * *p* < 0.05, ** *p* < 0.01, *** *p* < 0.001, **** *p* < 0.0001 vs. Aβ42 group (*n* = 8 mice/ group).

To test the in vivo effects of GAA, aggregated Aβ42 was administered i.c.v. to 8-week-old mice [41].The experimental design of the animals is shown in Figure 4A. Mice were divided into five groups: Sham (i.c.v. injection of normal saline and treatment with 0.1% DMSO-CMCNa, intragastrically (i.g.)); Aβ42-only (i.c.v. injection of Aβ42 and treatment with 0.1% DMSO-CMCNa, i.g.); Aβ42-Rog (i.c.v. injection of Aβ42 and treatment with 10 mg/kg Rog, i.g.); Aβ42-GAA (i.c.v. injection of Aβ42 and treatment with 100 mg/kg GAA, i.g.); and Aβ42-GAA-R428 (i.c.v. injection of Aβ42 and treatment with 25 mg/kg R428, and 100 mg/kg GAA, i.g.). Each agent was administered once daily for 16 days, and object-recognition and MWM tests were performed to evaluate the effect of GAA on Aβ42-treated mice.

For the object-recognition test, the discrimination index was used to evaluate learning and memory ability in mice, where a higher index value indicates a greater preference for the new object. The Sham group treated with vehicle preferred the novel objects, whereas the Aβ42-only group showed no significant preference. Administration of GAA and Rog effectively restored the impaired learning and memory abilities caused by Aβ42 injection, with the discrimination values significantly higher than those of the Aβ42- only group (Figure 4B,C). In the MWM test, the Aβ42-only group displayed significantly longer escape latency as compared with the Sham group during spatial acquisition training, which was improved by GAA or Rog treatment (Figure 4D). In the spatial probe test, the distance and time in the target quadrant, and the number of platform crossings in the Aβ42-only group, were significantly lower than those of the Sham group, indicating the impairment of spatial memory capacity, which was reversed by GAA or Rog treatment (Figure 4F–H). Moreover, the Aβ42 level in the hippocampus was downregulated by GAA treatment (Figure 4I), whereas IHC assessment, showed upregulated expression of LC3B in the hippocampus following GAA treatment (Figure 4J). Additionally, strong activation of microglia, which are Iba-1+ cells [42], in the hippocampus was observed in the Aβ42-only group, with this ultimately reversed by GAA treatment (Figure S3A). These findings indicated that GAA was beneficial to improving the inflammatory response caused by abnormal activation of microglia.

The results demonstrated that GAA ameliorated cognitive deficits in Aβ42-treated mice. The effect of GAA in Aβ42-treated mice was blocked by the Axl-specific inhibitor, R428 (Figure 4B–J and Figure S3A), consistent with the in vitro study. Taken together, these results indicated that GAA ameliorated cognitive deficits and decreased the accumulation of Aβ42 accumulation by promoting autophagy in an Axl-dependent manner.

## 3. Discussion

AD is a common neurodegenerative disease and among diseases with the highest incidence in the elderly population. It has become the third leading cause of death in the elderly [43]. Since 2003, apart from the approval of memantine complex to treat moderate-to-severe AD [44], there has been no effective drug available. Numerous studies have shown that abnormal production and accumulation of Aβ in nerve cells is sufficient to cause dysfunction of nerve cells and synapses [45]. Moreover, the Aβ polymer released outside of nerve cells induces neuroinflammation and causes more serious AD symptoms [46]. Aβ plaques as well as all types of Aβ oligomers, protofibrils, and fibrils were found in symptomatic AD as well as in pathologically defined preclinical AD (preAD) cases. However, amyloid plaques are not specific for symptomatic AD and can be seen in non-demented individuals [47,48]. For the neuropathological diagnosis of AD, all cases in which Aβ plaques are found in the brain are to be diagnosed with AD pathology regardless of their clinical status [49]. This definition does not imply that all of these cases would have necessarily converted into symptomatic AD but rather describes that non-demented patients can have AD pathology. In clinical trials, inhibitors and antibodies mainly targeting Aβ production, such as the γ-secretase inhibitor (semagacestat) and mAb drugs (bapineuzumab and gantenerumab), failed in phase III [50,51,52]. Despite its inhibition of Aβ production, semagacestat did not improve the cognitive status of patients, and there was even significant worsening of functional ability at higher doses [50].

Aβ mAb drugs form Aβ antibody complexes in brain tissue and cerebrospinal fluid that can potentially hinder the outflow of Aβ from the brain. Therefore, although Aβ mAb drugs can slow down the Aβ deposition in brain tissue, they cannot remove Aβ plaques and have shown weak Aβ clearance ability in clinical trials [53,54]. Because patients with advanced AD show greater propensity impaired clearance rather than excessive production of Aβ [6], it is preferable to remove the abnormal accumulation of Aβ in and outside of nerve cells in a timely and effective manner.

There have been great advances in research on *G. lucidum;* the main chemical components include polysaccharides, triterpenoids, amino acids, and organic germanium [55]. Modern pharmacological studies reveal that Ganoderma exhibits anti-tumor effects, can regulate the immune system, and is effective for treating cardiovascular and cerebrovascular diseases [56,57,58]. Additionally, Ganoderma has received attention for the treatment of neurological diseases. GLPs enhance the activation of fibroblast growth factor receptor 1, and its downstream extracellular signal-regulated kinase and AKT cascades, promote the proliferation of neuropodocytes in AD mice, and reduce cognitive deficits [27]. Another study showed that *G. lucidum* water extract can reduce the neuronal damage caused by Aβ by weakening the phosphorylation of c-Jun *n*-terminal kinase, c-Jun, and p38 mitogen-activated protein kinase, thereby preserving synaptic density and synaptic vesicle proteins [59]. Chemical structures of *G. lucidum* triterpenoids are based on lanostane, which is a metabolite of lanosterol whose biosynthesis is based on squalene cyclization [60]. It is more than obvious that the triterpene structure of ganoderic acids plays a vital part in their biological action. The activity of ganoderic acids could be mainly related to the hydroxylation of their lanostane triterpene structure [61]. Structurally, the polysaccharides of *G. lucidum* mostly comprise high molecular weight heteropolymers, where the major component is glucose, but also including xylose, mannose, galactose, and fructose [62]. This is probably the reason why GLPs does not have beneficial effects as those observed with GLTs. In the present study, we found that intracellular Aβ42 levels were significantly reduced by GLT treatment in BV2 cells. GAA is a major component of GLTs, and we also found that intracellular Aβ42 levels were significantly reduced by GAA treatment in BV2 cells, but not by GAD or GAG treatment. These findings point to GAA as a potential drug for the treatment of AD by virtue of its ability to clear Aβ.

In AD patients and animal models, microglia accumulate around senile plaques [63], constantly transform morphological perception, protect the intercellular environment, migrate to the abnormal position of intercellular substances, and become activated [64]. After activation, microglia are able to clear extracellular Aβ through phagocytosis [65], thereby reducing accumulation of the Aβ protein between the gaps. Autophagy is an essential degradation pathway that removes abnormal protein aggregates and is responsible for protein homeostasis and neuronal health [66]. Autophagy plays an important role in Aβ production and metabolism, and the assembly of tau; thus, its dysfunction can potentially lead to AD progression [67,68]. In the present study, we found that the ability of GAA to clear Aβ was mainly dependent on promoting autophagy, but not ADEs. Based on these findings, autophagy might represent a new target for the development of drugs for AD. In fact, to date, many autophagy modulators have shown positive effects in AD treatment [69].

The TAM receptor, tyrosine kinase Axl, can regulate microglial functions such as responding to inflammation in the CNS and clearing misfolded proteins [70]. Moreover, upregulation of Axl in microglia promotes plaque clearance [71]. Therefore, the Axl receptor in microglia might be a potential target for alleviating AD symptoms. After interacting with GAS6, Axl induces autophagy in macrophages [21]. Additionally, Axl activates Rac1. Based on existing evidence that Rac1 improves stroke symptoms through Pak1 signaling, the role of Pak1 in autophagy initiation might be through Axl.

ATG5, BECN1, and MAP1LC3B proteins are essential for inducing autophagy; therefore, we monitored their corresponding mRNA transcripts by qRT-PCR after treating BV2 cells with GAA. The transcript levels of these autophagy genes were increased in GAA-treated BV2 cells after 3 h, 6 h, and 12 h; however, these increases were reduced at 24 h post-treatment (Figure 2C–E). This trend might be related to Axl activation. A previous study reported that the expression of autophagy-related genes caused by Axl activation with GAS6 showed a similar pattern of changes [21]. Additionally, immunofluorescence and Western blot analyses showed that GAA did not inhibit the lysosomal activity of BV2 cells (Figure 3G and Figure S3A). Taken together, these results indicated that GAA mediates autophagy induction by increasing the expression of *Atg5*, *Becn1*, and *Map1lc3b* in BV2 cells. While originally identified as a cell survival mechanism, autophagy plays highly context-specific roles in mediating cell death. As discussed recently, autophagy may mediate certain cell death processes [72]. In *Bax/Bak*-deficient mouse embryonic fibroblasts, autophagy is required for cell death induced by chemotherapeutic drugs, which is blocked by genetically inhibiting autophagy [73].

The increased LC3B-II level observed following GAA treatment was not caused by blocking autophagosome degradation. We found that the promotion of Aβ42 degradation by GAA in lysosomes was prevented by the Axl antagonist R428 and the Pak1 inhibitor IPA-3 in BV2 cells. In the object-recognition and MWM test, GAA administration effectively restored the preference for novel objects, shortened escape latency, and increased the distance and time in the target quadrant and the number of platform crossings in Aβ42-injected mice (Figure 4B–H), indicative of memory recovery. Rog, a peroxisome proliferator-activated receptor γ (PPAR-γ) agonist and anti-diabetic agent, may improve symptoms of AD through promotion of Aβ clearance [74]. In the Aβ-injected mice, Rog was used as a positive drug to ameliorate Aβ42-induced behavioral deficits. These effects were consistent with the downregulation of Aβ42 levels in the hippocampus (Figure 4I). Notably, there was a negative correlation between Aβ42 and LC3B levels in the hippocampus (Figure 4I–J). Furthermore, the effect of GAA in Aβ42-treated mice was blocked by the Axl-specific inhibitor, R428 (Figure 4B–J), consistent with the in vitro study. These results suggest a positive feedback loop between the Axl and Pak1 signaling pathways to activate autophagy, which might explain the notable high-efficiency clearance of Aβ to improve AD symptoms following GAA treatment.

**Figure 5:**
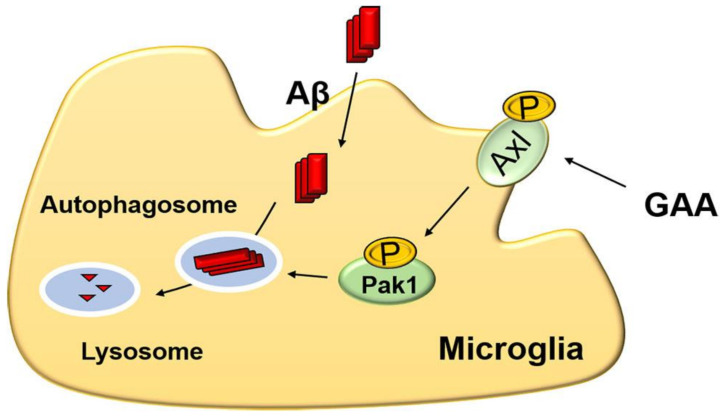
Proposed mechanism of GAA-mediated Aβ clearance in vitro.

In conclusion, this study showed that GAA significantly increased autophagy in BV2 cells by enhancing Axl phosphorylation, and that GAA treatment effectively promoted Aβ42 clearance by microglia and ameliorated cognitive deficits in AD mouse models. Additionally, we found that GAA upregulated the level of autophagy, at least in part, by activating Pak1 through Axl in BV2 cells (Figure 5). These findings offer critical insight into the mechanism by which the spore powder of *G. lucidum* may be effective in treating AD [75].

## 4. Materials and Methods

### 4.1. Reagents

Human Aβ42 peptides and FITC-labeled human Aβ42 peptides were purchased from China Peptides (Suzhou, China). GAA, ganoderic acid D (GAD), and ganoderic acid G (GAG) were purchased from Pufeide Biological Technology Co. Ltd. (Chengdu, China). Rosiglitazone (S2556) was purchased from Selleck (Houston, TX, USA). Enalapril maleate (HY-B0331A), sacubitrilat (HY-17620), arachidonic acid (HY-109590), EACC (HY-129111), chloroquine (HY-17589A), recilisib (HY-101625), R428 (HY-15150), and IPA-3 (HY-15663) were purchased from MedChem Express (Shanghai, China). Stock solutions of all drugs were made with dimethyl sulfoxide (DMSO; Sigma-Aldrich, St. Louis, MO, USA) and diluted in Dulbecco’s modified Eagle medium (DMEM) to final concentrations for cell experiments or diluted in saline for animal treatments. The final concentration of DMSO was ≤0.1%. The purity of each compound was determined to be >98% by high performance liquid chromatography. DMEM, fetal bovine serum (FBS), and penicillin/streptomycin were purchased from Gibco (Grand Island, NY, USA). The human Aβ42 enzyme-linked immunosorbent assay (ELISA) kit was purchase from Cusabio (Wuhan, China). Lyso-Tracker Red (C1046) was purchased from Beyotime (Shanghai, China). Carboxymethylcellulose sodium (CMCNa) was purchased from Sigma-Aldrich (c5678, St. Louis, MO, USA).

### 4.2. Antibodies

The rabbit monoclonal antibody (mAb) targeting light-chain 3B (LC3B; A19665), the lysosomal-associated membrane protein 1 (LAMP1) rabbit polyclonal antibody (pAb) (A16894), pan-Akt rabbit pAb (A18120), PAK1 rabbit mAb (A19608), phosphorylated (phospho)-PAK1/2/3-S144/S141/S139 rabbit mAb (AP1158), glyceraldehyde 3-phosphate dehydrogenase (GAPDH) rabbit mAb (A19056), and Iba1 rabbit pAb (A1527) antibodies were purchased from Abclonal (Wuhan, China). Phospho-Akt (Ser473) rabbit mAb (4060), Axl Rabbit mAb (8661), Phospho-Axl (Tyr698) Rabbit mAb (44463), Sequestosome 1 (SQSTM1/p62) Rabbit mAb (39749) antibodies were purchased from Cell Signaling Technology (Danvers, MA, USA). Horseradish peroxidase (HRP)-labeled goat anti-rabbit or mouse secondary antibodies were purchased from Beyotime (Shanghai, China).

### 4.3. Instrumentation and Chromatographic Separation Conditions

#### 4.3.1. Liquid Chromatography Conditions

ACQUITY UPLC/quadruple time-of-flight mass spectrometry systems (Waters Corp., Milford, MA, USA) was used in this study. Chromatographic separation was performed at 25 °C on an ACQUITY UPLC-BEH-C18 column (100 × 2.1 mm, 1.7 μm; Waters Corp.). The mobile phase comprised of 0.1% acetic acid and water (A) and acetonitrile (B). The gradient elution is shown in Table S1.

#### 4.3.2. Experimental Conditions for Mass Spectrometry (MS)

For mass analysis, the centroid mode was adopted, and the mass range set at 100 to 1500 mass-to-charge ratios (*m*/*z*) in negative ionization modes. The parameters for the electrospray ionization source were as follows: flow rate of nebulizer gas (N_2_), 50 L/h; flow rate of desolvation gas (N_2_), 600 L/h; desolvation temperature, 300 °C; source temperature, 120 °C; capillary voltage, 3000 V; sample cone, 30 V; and extraction cone voltage, 4.0 V. For tandem MS (MS/MS) experiments, variable collision energy (20–50 eV) was optimized for individual compounds.

### 4.4. Cell Culture

BV2 cells were grown in DMEM supplemented with 10% FBS and 1% penicillin/streptomycin with isobaric oxygen in 5% CO_2_ at 37 °C.

### 4.5. Total Protein Quantification Assay

Total protein quantification assay was performed according to BCA Protein Assay Kit (Beyotime, Shanghai, China): 20 μL of each standard or sample replicate was pipetted into a microplate well (Thermo Scientific™ Pierce™ 96–Well Plates, Product No. 15041). BCA working solution was prepared by mixing 50 parts of BCA Reagent A with 1 part of BCA Reagent B (50:1, Reagent A:B). 200 μL of BCA working solution was added to each well. The plate was incubated at 37 °C for 30 min. The absorbance was measured at 562 nm on a plate reader. The average 562 nm absorbance measurement of the Blank standard replicates were subtracted from the 562 nm measurements of all other individual standard and sample replicates. A standard curve was prepared by plotting the average Blank–corrected 562 nm measurement for each BSA standard vs. its concentration in μg/mL. The standard curve was used to determine the protein concentration of each sample.

### 4.6. Intracellular Aβ-Clearance Assay

The intracellular Aβ clearance assay was performed as previously described [76]. Briefly, BV2 cells were administrated with 2 μM soluble Aβ42 in serum-free medium for 24 h in the presence of drugs. At the end of the treatment, cells were washed with phosphate-buffered saline (PBS) to remove remaining Aβ42 was that attached on the cell surface. Then, cells were lysed by 1% sodium dodecyl sulfate (SDS) and the intracellular Aβ42 levels were measured by the ELISA kit, according to the manufacturer’s instructions.

Aβ42 quantification was performed according to the Human Aβ42 enzyme-linked immunosorbent assay (ELISA) kit (Cusabio, Wuhan, China): Reagents, samples, and standards were prepared as instructed. 100 μL standard or sample was added to each well and the plate was incubated for 2 h at 37 °C. The liquid in each well was removed. 100 μL Biotin-antibody (1*) was added to each well and the plate was incubated for 1 h at 37 °C. Each well was washed 3 times. 100 μL HRP-avidin (1*) was added to each well and the plate was incubated for 1 h at 37 °C. Each well was washed 5 times. 90 μL TMB substrate was added to each well and the plate was incubated 30 min at 37 °C, protected from light. 50 μL Stop Solution was added to each well and the absorbance was read at 450 nm within 5 min. The standard curve was used to determine the Aβ42 concentration of each sample. The micrograms of proteins used in Aβ42 quantification in cells were from 150 ug to 230 ug. The micrograms of proteins used in Aβ42 quantification in tissues were from 190 ug to 240 ug.

### 4.7. Flow Cytometry

Microglial BV-2 cells were plated at a density of 10^5^ cells/well in a six-well plate overnight in DMEM containing 10% FBS. After 24 h, the media was replaced with DMEM containing 1% FBS in the presence of FITC-labeled Aβ42 (1 µM), Rog (10 µM), and GLT or GAA at indicated concentrations for 24 h. Cells were washed with PBS and collected for analysis by flow cytometry with a BD Accuri C6 flow cytometer (BD Biosciences, New York, NJ, USA). The fluorescence value of 10,000 cells was detected in each group.

### 4.8. Western Blot Assay

Cells were lysed on ice with radioimmunoprecipitation assay (RIPA) buffer [50 mM Tris (pH7.4), 150 mM NaCl, 1% NP-40, 0.5% sodium deoxycholate, 0.1% SDS, and 0.5 mM EDTA] with a protease inhibitor and phosphatase inhibitors (Roche, Mannheim, Germany). Protein samples were separated by electrophoresis using 10% or 15% SDS polyacrylamide gel electrophoresis and transferred onto nitrocellulose membranes, which were blocked with 5% non-fat milk for 1 h at room temperature and incubated with primary antibodies overnight at 4 °C. (LC3-I and LC3-II were separated from each other using gels of 15% polyacrylamide.) The membranes were then washed and incubated with secondary antibodies for 2 h at room temperature and developed using enhanced chemiluminescence detection. Signals were detected with a Tanon-5200 chemiluminescent imaging system (Tanon, Shanghai, China), and protein levels were analyzed using Image J (NIH, Bethesda, MD, USA) and normalized to the corresponding GAPDH level.

### 4.9. Quantitative Reverse Transcription (qRT)-PCR

Total RNA was extracted from BV2 cells using TRIzol reagent (Life Technologies, Carlsbad, CA, USA) according to the manufacturer’s instructions. RNA concentrations were equalized and converted to cDNA using the Hiscript reverse transcriptase kit (Vazyme, Nanjing, China). Gene expression was measured using qPCR system with SYBR-green (Roche, Basel, Switzerland) and normalized against *Gapdh*. The primer sequences are shown in Table S2.

### 4.10. Intracellular Localization of Aβ42

Briefly, cells were incubated with FITC-conjugated-Aβ42 for 6 h, followed by incubation with a lysosome tracker for 0.5 h. Immunofluorescence was visualized and captured by confocal microscopy (LSM 710; Zeiss, Oberkochen, Germany).

### 4.11. Animal Study

All experiments and animal care in this study were conducted in accordance with the National Institutes of Health Guide for the Care and Use of Laboratory Animals (NIH Publication No. 8023, revised 1978), and the Provision and General Recommendation of Chinese Experimental Animals Administration Legislation and approved by the Science and Technology Department of Jiangsu Province [SYXK (SU) 2016-0011]. Male C57BL/6 J (specific pathogen-free, 6-week old, and 20–24 g) were purchased from Nanjing University (Nanjing, China). The animals were kept under a constant temperature (24 °C) with a 12 h light/dark cycle and fed with standard food pellets with access to sterile water ad libitum. Mice were randomly divided into the following groups (*n* = 8 mice/group) [77]: Sham + CMCNa, Aβ42 injection + CMCNa, Aβ42 injection + rosiglitazone (Rog), Aβ42 injection + GAA, and Aβ42 injection + GAA + R428. CMCNa (0.1% DMSO), Rog (10 mg/kg/d), GAA (100 mg/kg/d), or R428 (25 mg/kg/d, 30 min before GAA administration) + GAA (100 mg/kg/d) was administered to mice by oral gavage once daily for 16 days.

#### 4.11.1. Preparation of the AD Model

For i.c.v. injection of soluble aggregated Aβ42, mice were anesthetized with pentobarbital sodium (50 mg/kg, intraperitoneal injection), and then placed in a stereotactic apparatus (Motorized Stereotaxic Stereo Drive; Neuronetics, Malvern, PA, USA). Freshly prepared Aβ42 (82 pmol/µL in 0.1 M PBS) was injected into the bilateral ventricles (0.3 mm posterior, 1.0 mm lateral, and 2.5 mm ventral to bregma) using a stepper-motorized micro-syringe fitted with a 26-gauge needle at a rate of 0.2 µL/min. The total injection volume per mouse was 5 µL. Mice infused with vehicle at the same volume served as the control group (Sham) [41].

#### 4.11.2. MSM Test

The MWM test was performed to detect spatial memory as previously described [78]. The escape latencies, time spent in target quadrant, and platform-crossing times were recorded and analyzed by the analysis-management system (Viewer 2 Tracking Software; Shanghai, China).

#### 4.11.3. Object-Recognition Test

The object-recognition test was performed as described previously [79]. Briefly, the test proceeded in a square open field apparatus with a side length of 50 cm. During the habituation session, each mouse was individually placed into the empty open field, facing the wall nearest the operator and allowed to explore the open field for 6 min. The familiarization session was performed 24 h after the habituation step. Two cylinders were placed in the open field. The mouse was placed in the open field with its head positioned opposite the objects. The mouse was allowed to freely explore for 10 min and then return to its home cage. In the experiments, one cube and one cylinder were used during the test session. The test session was performed 24 h after the familiarization session. The two objects were placed at the same location as before and the animals were allowed to explore freely for 6 min. The exploration time required to identify the familiar object and novel object was recorded for analysis. The discrimination index was calculated as follows: Discrimination index = (% time with novel object − % time with familiar object)/(% time with novel object + % time with familiar object). The behavior parameters were automatically measured using ANY-maze video tracking software (Stoelting Co., Kiel, WI, USA).

#### 4.11.4. Immunohistochemical (IHC) Assessment

Whole brain tissue was fixed in 4% paraformaldehyde at 4 °C for 8 h, taken out in a 70% ethanol solution for 5 min, then placed in 80%, 90%, 95%, and absolute ethanol for gradient dehydration for 4 h each time, respectively; finally, tissues were immersed in xylene for 30 min, and then embedded in paraffin and sectioned using a microtome, with the slices subsequently stained with hematoxylin. To detect the expression of LC3B or Iba-1, rabbit anti- LC3B or anti-Iba-1 pAb (1:100), HRP-labeled goat anti-rabbit antibodies (Boster, Wuhan, China), and diaminobenzidine for chromogenic reaction were applied. Images were obtained under a microscope (IX53; Olympus, Tokyo, Japan) to analyze LC3B or Iba-1 expression in the hippocampus.

#### 4.11.5. Brain Aβ42 Measurement

Hippocampus samples were lysed in RIPA buffer containing a protease inhibitor (Roche, Mannheim, Germany), and Aβ42 levels were detected using an ELISA kit and normalized against total protein concentration.

### 4.12. Statistical Analysis

Data were expressed as the mean ± standard deviation. Comparisons between two groups were assessed using a Student’s *t*-test, and comparisons between three or more sets of data were made using one-way or two-way analysis of variance followed by Dunnett’s post hoc test with GraphPad Prism (v.8.0.2; GraphPad Software, La Jolla, CA, USA). Differences with a *p* < 0.05 were considered significant.

## Data Availability

Not applicable.

## Supplementary Materials

The following are available online at https://www.mdpi.com/article/10.3390/ijms22115559/s1. Table S1.
