# Supplementary material for: Ganoderic Acid A Promotes Amyloid-β Clearance (In Vitro) and Ameliorates Cognitive Deficiency in Alzheimer’s Disease (Mouse Model) through Autophagy Induced by Activating Axl
Source: Int J Mol Sci. 2021 May 24;22(11):5559. doi: 10.3390/ijms22115559 (PMC8197357; doi:10.3390/ijms22115559)
Supplement: Supplementary file 1 [file ijms-22-05559-s001.zip › Supplementary Information.pdf]

Supplementary Information

Ganoderic acid A promotes amyloid-β clearance (*in vitro*) and ameliorates cognitive deficiency in Alzheimer's disease (mouse model) through autophagy induced by activating Axl

Authors: Lifengrong Qi [1], Shuai Liu[1, 2], Yuci Liu[1], Ping Li[1] and Xiaojun Xu[1, 2] *

Running title: Ganoderic acid A activates autophagy to ameliorate cognitive deficiency

Affiliations:

1 State Key Laboratory of Natural Medicines, China Pharmaceutical University, 210009, Nanjing, Jiangsu, China.

2 Jiangsu Key Laboratory of Drug Discovery for Metabolic Diseases, China Pharmaceutical University, 210009, Nanjing, Jiangsu, China.

Corresponding author:

Xiaojun Xu, State Key Laboratory of Natural Medicines, Jiangsu Key Laboratory of Drug Discovery for Metabolic Diseases, China Pharmaceutical University, 210009, Nanjing, Jiangsu, China. Telephone number: +86-2583271203, E-mail: xiaojunxu2000@163.com.

Table S1. The gradient elution used for liquid chromatography.

Time (min)	Flow (mL/min)	%A	%B
Initial	0.2	90	10
10	0.2	70	30
15	0.2	60	40
20	0.2	20	80
25	0.2	5	95
28	0.2	5	95
30	0.2	90	10
35	0.2	90	10

Table S2. The primer sequences used for qRT-PCR.

Gene		Sequence (5' to 3')
Atg5	F	AAGTCTGTCCTTCCGCAGTC
	R	TGAAGAAAGTTATCTGGGTAGCTCA
Becn1	F	AGGATGGTGTCTCTCGAAGATT
	R	GATCAGAGTGAAGCTATTAGCACTTTC
Mapllc3b	F	CCCCACCAAGATCCCAGT
	R	CGCTCATGTTCACGTGGT
Gapdh	F	ATGGTGAAGGTCGGTGTGAA
	R	GGTCGTTGATGGCAACAATCTC

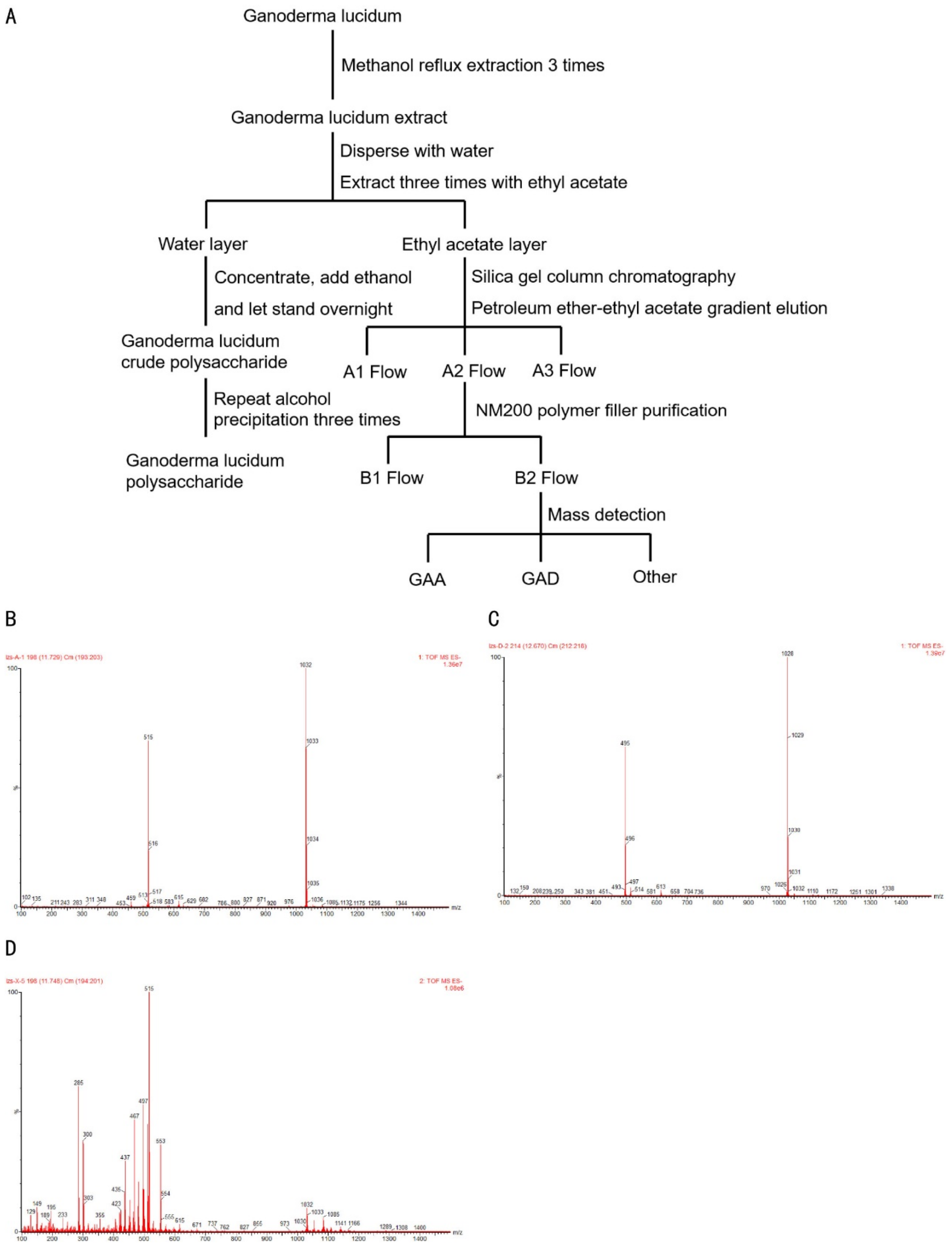

Figure S1. Extraction of triterpene acids and polysaccharides from *Ganoderma lucidum* and MS analysis of the triterpene acids. **(A)** Flow chart of Ganoderma extraction. **(B-D)** GAA, GAD and other ingredients detected by MS.

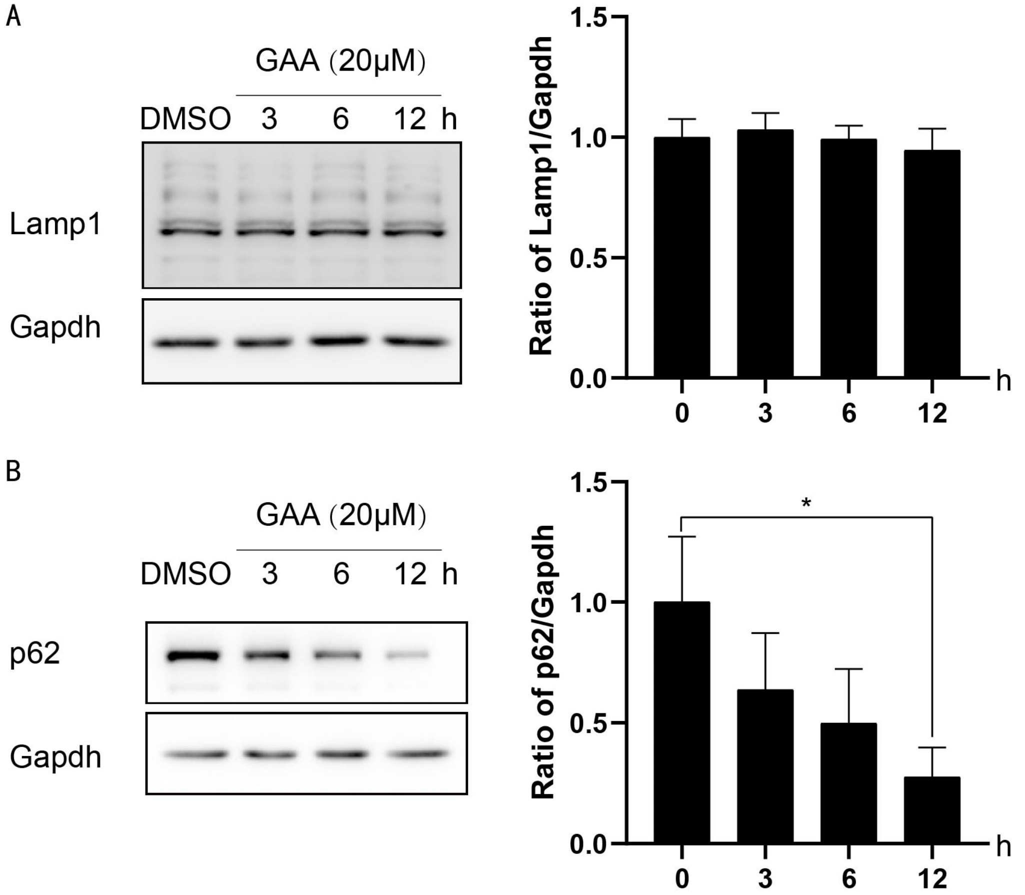

Figure S2. LAMP1 and p62 level in GAA-treated microglial cells. **(A)** LAMP1 level (N =3) and **(B)** p62 level (12 h vs 0 h: p = 0.0138; N = 3) in GAA-treated microglial cells detected by western blot. N is the number of replicates (biological and technical) used for each of the described results. *$p < 0.05$ vs. indicated control.

A

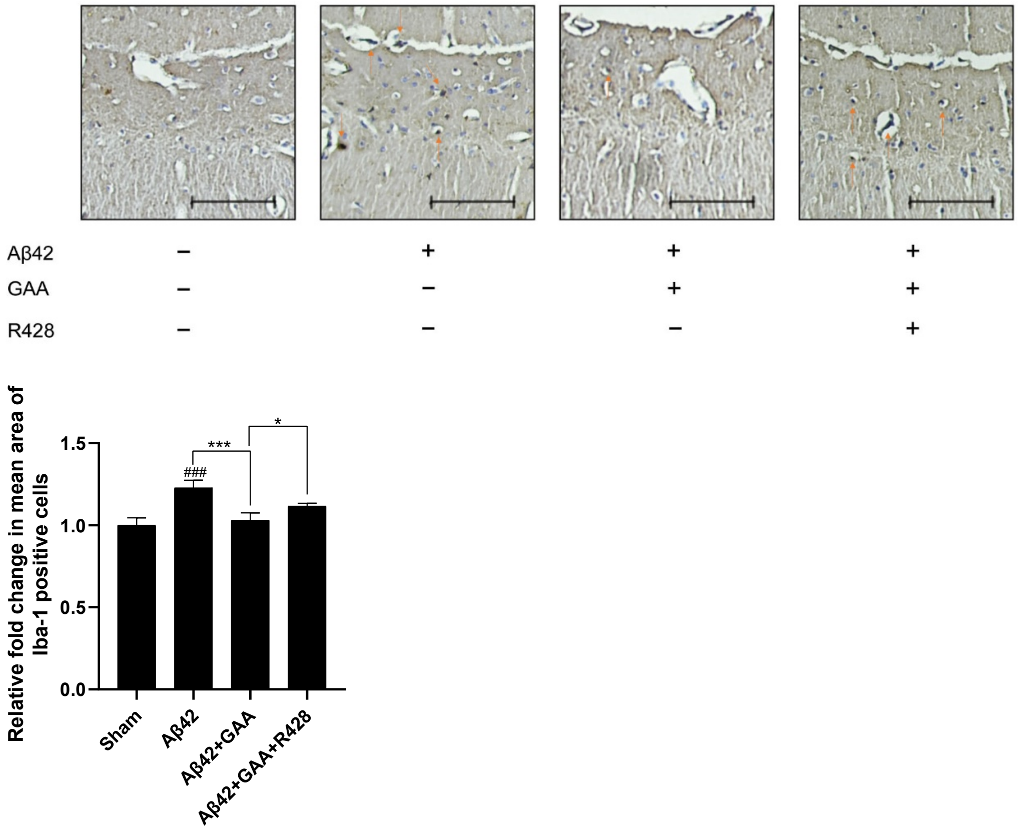

Figure S3. GAA relieves over-activation of microglia in the hippocampus. **(A)** Iba-1 level in the hippocampus detected by IHC assessment. Images were obtained under a microscope (Scale bar: 100 μm). Aβ42 vs Sham: $p = 0.0004$; Aβ42 + GAA vs Aβ42: $p = 0.0009$; Aβ42 + GAA + R428 vs Aβ42 + GAA: $p = 0.0118$; N = 4. N is the number of replicates (biological and technical) used for each of the described results. ###$p < 0.001$ vs. Sham group. * $p < 0.05$, **$p < 0.01$, ***$p < 0.001$, ****$p < 0.0001$ vs. Aβ42 group.
