# Supplementary figures and images for: Ganoderic Acid A Promotes Amyloid-β Clearance (In Vitro) and Ameliorates Cognitive Deficiency in Alzheimer’s Disease (Mouse Model) through Autophagy Induced by Activating Axl
Source: Int J Mol Sci. 2021 May 24;22(11):5559. doi: 10.3390/ijms22115559 (PMC8197357; doi:10.3390/ijms22115559)

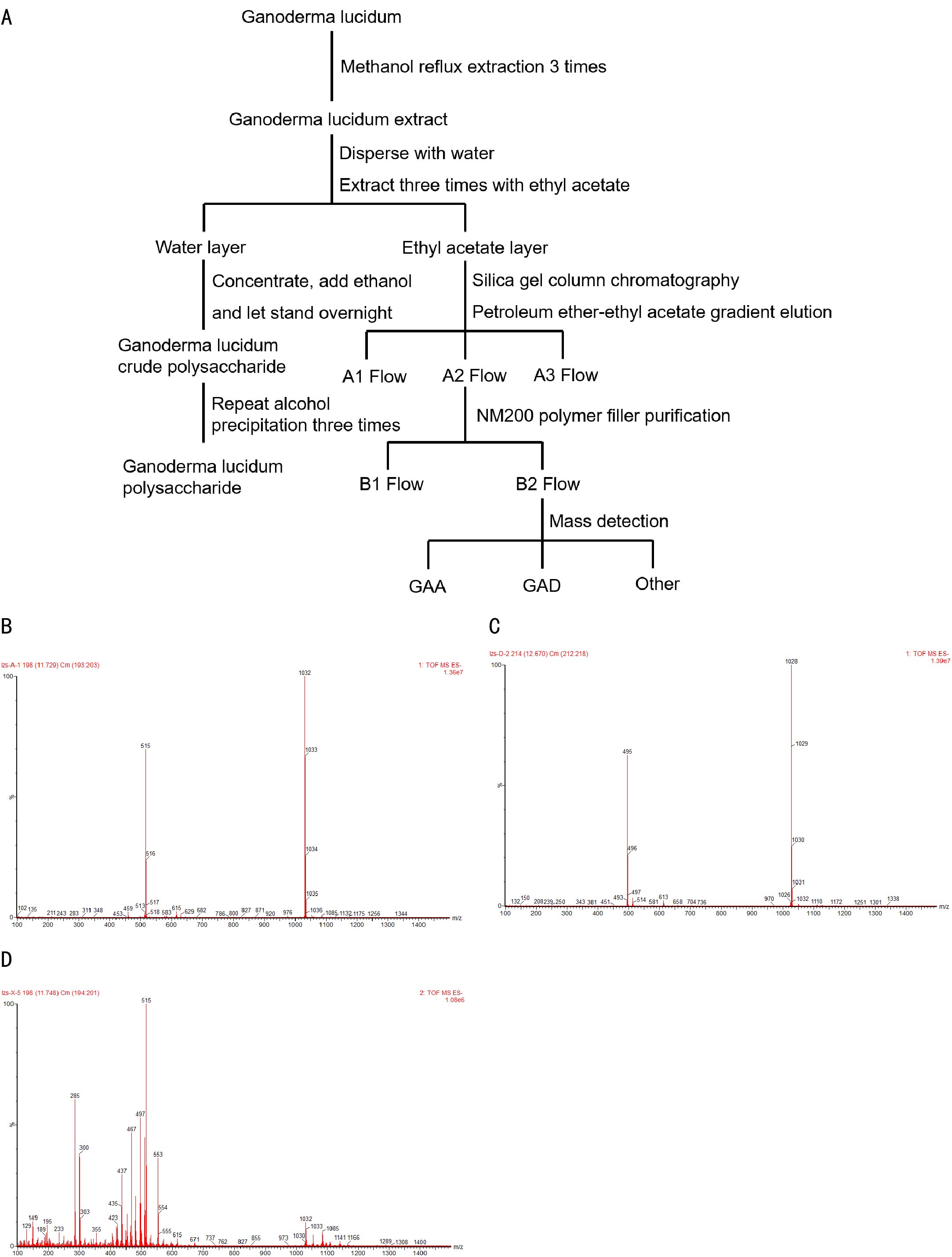

Supplement: Supplementary file 1 [file ijms-22-05559-s001.zip › FigS1.jpg]

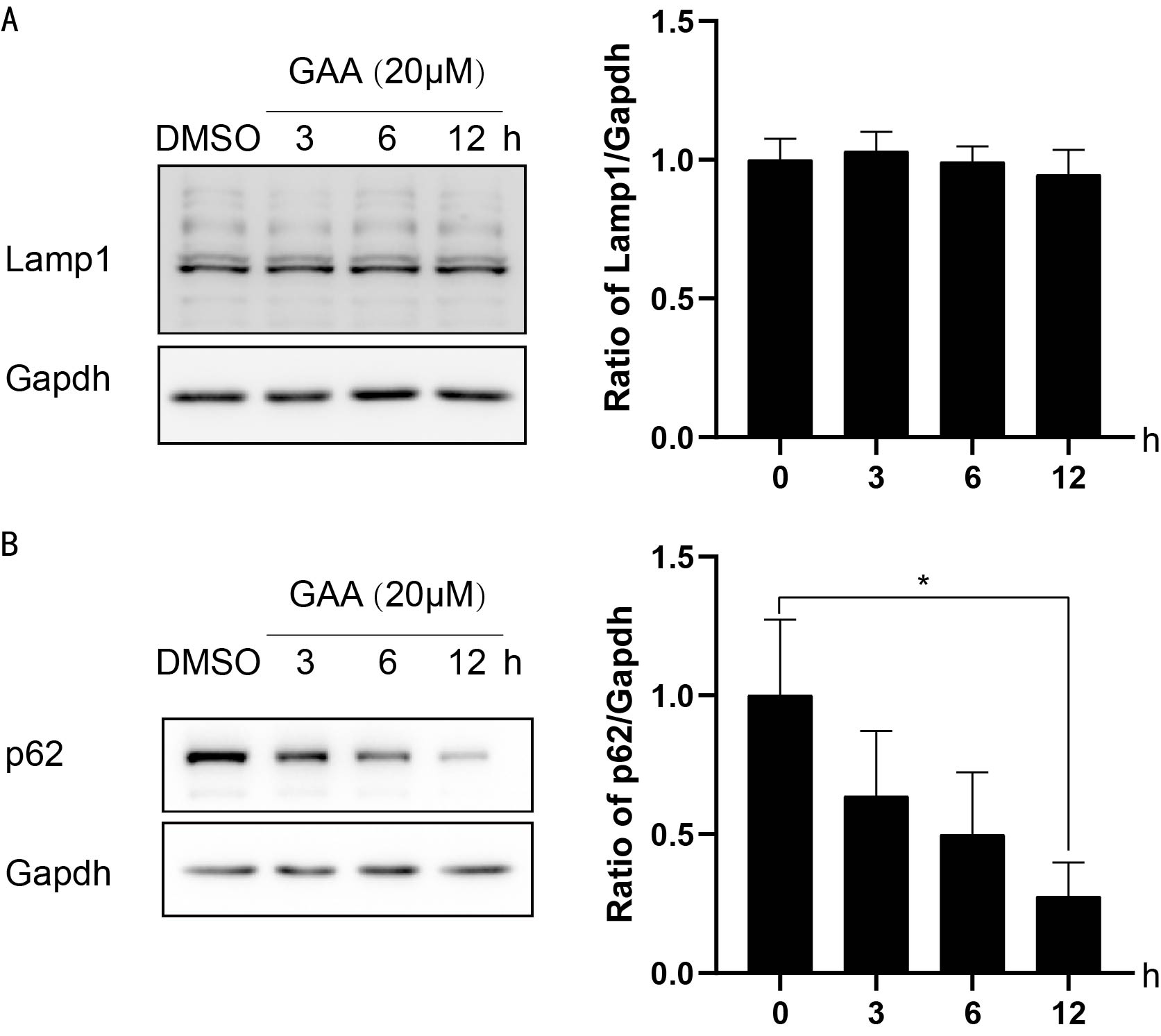

Supplement: Supplementary file 1 [file ijms-22-05559-s001.zip › FigS2.jpg]

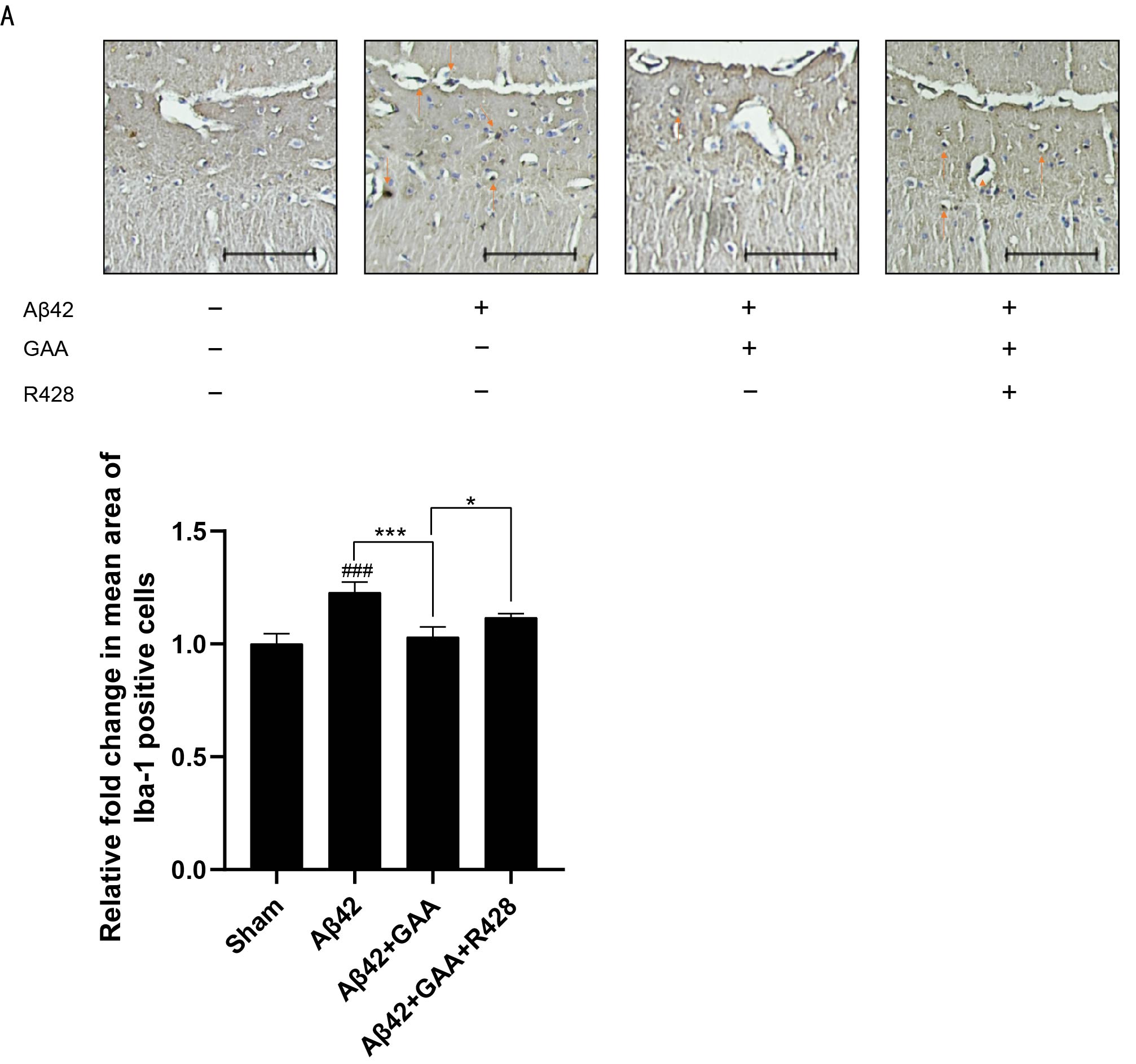

Supplement: Supplementary file 1 [file ijms-22-05559-s001.zip › FigS3.jpg]
